# From By-Product to Bioactive: New Antioxidant and Bioavailable Peptides Derived from Milk Permeate Targeting the Nrf2/Keap1 Pathway in Intestinal Cell Models

**DOI:** 10.3390/antiox15050527

**Published:** 2026-04-22

**Authors:** Valeria Scalcon, Alessandro Grinzato, Federico Fiorese, Alessandra Folda, Stefania Ferro, Gianfranco Betti, Marco Bellamio, Emiliano Feller, Oriano Marin, Maria Pia Rigobello

**Affiliations:** 1 Department of Biomedical Sciences, University of Padova, via Ugo Bassi 58/b, 35132 Padova, Italy; 2 Centrale del Latte d’Italia S.p.A., Sede di Firenze, via dell’Olmatello 20, 50127 Firenze, Italy; 3 Centrale del Latte d’Italia S.p.A., Sede di Vicenza, via Faedo 60, 36100 Vicenza, Italy

**Keywords:** bioactive peptides, antioxidants, milk permeate, oxidative stress, Nrf2, bioavailability

## Abstract

This study investigates the antioxidant properties of several synthetic peptides derived from milk proteins previously identified in milk permeate, a by-product of the dairy industry. The aim of the research is to identify which peptides present in milk permeate are responsible for its antioxidant activity. A comprehensive experimental strategy was employed to evaluate their antioxidant potential, including in silico selection, in vitro free radical scavenging assays and cellular models using Caco-2 and HCT116 cell lines. The peptides were screened using a molecular docking approach for their potential interaction with the Kelch-like ECH-associated protein 1/nuclear factor erythroid 2-related factor 2 (Keap1/Nrf2) pathway, and eight out of twenty-eight were selected and synthesized for further analyses. In vitro, six of the selected peptides demonstrated significant direct antioxidant activity in the DPPH scavenging assay, and two in the ABTS scavenging test. In cellular environments, three peptides (LPAPELGPRQA, LPIIQKLEPQI and NGQVWEESLKRL) effectively protect cells from oxidative stress induced by *tert*-butyl hydroperoxide, reducing reactive oxygen species production and partially mitigating lipid peroxidation. Further investigation showed that two of them (LPAPELGPRQA and LPIIQKLEPQI) effectively induce the Keap1/Nrf2 pathway, as evidenced by a ∼1.5-fold increase in Nrf2 levels and overexpression of downstream proteins. Permeability studies revealed that these peptides can cross the intestinal monolayer (2–3% in 2 h), suggesting potential systemic effects. Overall, these findings highlight the multifunctional antioxidant properties of the investigated peptides and support their potential application as nutraceuticals or therapeutic agents for oxidative stress-related conditions.

## 1. Introduction

Bioactive peptides derived from milk have garnered increasing attention due to their potential health benefits, particularly in the context of functional foods and nutraceuticals [1,2,3]. These peptides, which are released during the enzymatic hydrolysis of milk proteins (both caseins and whey), exhibit positive effects on health-bearing activities such as antihypertensive, anti-inflammatory, analgesic and antioxidant [3,4,5]. Regarding the antioxidant activity, it can derive directly from free radical scavenging, or via metal ion chelation, inhibition of lipid peroxidation or effect on intracellular antioxidant pathways [6]. Among these pathways, the Kelch-like ECH-associated protein 1/nuclear factor erythroid 2-related factor 2 (Keap1/Nrf2) pathway is one of the major axes promoting the transcription of phase II and antioxidant proteins [7]. The dual action—both direct scavenging of free radicals and indirect modulation of antioxidant defenses—makes certain antioxidant peptides particularly effective in combating oxidative stress. The latter, which these peptides help to mitigate, is a major contributor to the development of chronic diseases such as cardiovascular diseases, cancer, diabetes, and neurodegenerative disorders. Thus, the identification of the peptides may be helpful to potentially utilize them in a nutraceutical intervention in the context of chronic diseases [8,9,10,11]. Identifying new bioactive peptides and understanding their specific mechanisms of action are essential steps in advancing the development of antioxidant functional foods. These efforts will not only enhance the nutritional value of food products but also provide targeted health benefits, offering a promising avenue for the prevention and management of various oxidative stress-related diseases.

In this context, we previously reported that peptide fractions derived from milk permeate, a dairy farm by-product, exhibited strong antioxidant properties, effectively scavenging reactive oxygen species (ROS), promoting Nrf2 transcriptional activity and protecting cells from oxidative damage in vitro [12]. Furthermore, an antioxidant effect was also observed in vivo on the zebrafish model, increasing the translational potential of our findings. With the present research, our aim is to identify which specific peptides present in the milk permeate-derived peptide fractions are responsible for the observed antioxidant activity. Indeed, as research progresses, the elucidation of the specific molecular mechanisms through which these peptides exert their effects is crucial for optimizing their use in health-promoting applications.

In addition, the bioavailability and absorption of these bioactive peptides in the gut are critical for their effectiveness in exerting systemic antioxidant effects. Studies have shown that after ingestion, these peptides can be absorbed intact or as smaller fragments through the intestinal epithelium via both passive and active transport mechanisms [2,13,14]. Once absorbed, these peptides can enter the bloodstream and reach target tissues, where they continue to exhibit their activity. The peptide structure, amino acid composition, and the presence of specific transporters in the gut all influence the rate and extent of their absorption. Thus, we decided to evaluate also the bioavailability of the peptides utilizing an in vitro Transwell^®^ model, which can mimic the intestinal epithelium.

## 2. Methods

### 2.1. Reagents

All chemicals and reagents, if not stated otherwise, were purchased from Merck-Fluka-Sigma-Aldrich (Darmstadt, Germany).

### 2.2. Molecular Docking Analysis

Using an in silico molecular docking approach, the peptides were assessed for their capacity to promote Nrf2/Keap1 dissociation by analyzing their potential binding to Keap1. The Kelch domain of Keap1, which interacts with the conserved DxETGE motif located in the Neh2 domain of Nrf2 (PDB 2FLU), was chosen as a target for the docking analysis [15]. The Nrf2 peptide present in the crystal structure was removed, and the resulting model was equilibrated at 300 K and protonated to a pH of 7.4. The peptide–protein interaction was assessed using two different docking methods, CABS-Dock and GalaxyPepDock, simultaneously [16,17], running 10 replicas per peptide (access May 2023). The Docking simulation was performed using the full Kelch domain as the searchable surface (i.e., no grid box restriction) to avoid bias in binding site selection; however, in agreement with the internal binding score of each software, the peptides that bound with the Neh2 binding site of Nrf2 were preferred. The docking results were then analyzed using PISA (Proteins, Interfaces, Structures and Assemblies), and the peptides were scored based on the complex solvation free energy, the dissociation energy, and the solvation free energy *p*-value (mean value reported in Table S1) [18,19]. The protocol was validated using the native Nrf2 peptide from PDB 2FLU.

### 2.3. Peptide Synthesis

The synthesis of the eight peptides (Table 1, see below in the Results section) was carried out using a solid-phase peptide synthesis approach on an automated peptide synthesizer (Syro II, MultiSynTech GmbH, Witten, Germany). The peptide chains were assembled stepwise on Wang resins pre-loaded with the first N-α-Fmoc-protected amino acid, following the standard Fmoc protection strategy. HATU (Hexafluorophosphate Azabenzotriazole Tetramethyl Uronium) was employed as the coupling reagent throughout the synthesis. The following side-chain-protected amino acid building blocks were used: Fmoc-Glu(OtBu)-OH, Fmoc-Gln(Trt)-OH, Fmoc-Asn(Trt)-OH, Fmoc-His(Trt)-OH, Fmoc-Ser(tBu)-OH, Fmoc-Lys(Boc)-OH, Fmoc-Tyr(tBu)-OH, Fmoc-Arg(Pbf)-OH, Fmoc-Asp(OtBu)-OH, Fmoc-Trp(Boc)-OH and Fmoc-Thr(tBu)-OH. Following chain assembly, side-chain deprotection and resin cleavage were simultaneously achieved by treatment with a cocktail containing 88% (*v*/*v*) trifluoroacetic acid (TFA), 5% phenol (*w*/*v*), 5% H_2_O (*v*/*v*) and 2% triisopropylsilane (*v*/*v*) under shaking at room temperature for 2.5 h. The resin was subsequently removed via vacuum filtration, and the peptides were precipitated using cold diethyl ether followed by centrifugation. After two additional washing steps with cold diethyl ether, the crude peptides were purified by flash chromatography using a Biotage SP1 system equipped with a SNAP Ultra C18 12 g cartridge packed with Biotage HP-Sphere C18 25 μm spherical silica (Biotage, Uppsala, Sweden). Final confirmation of molecular mass was obtained via MALDI-TOF/TOF mass spectrometry (ABI 4800, AB Sciex, Framingham, MA, USA).

### 2.4. Evaluation of Antioxidant Activity with the ABTS^•+^ and DPPH Scavenging Assays

ABTS^•+^ was generated by reacting 7 mM ABTS (2,2′-azinobis(3-ethylbenzothiazoline 6-sulfonate)) with 2.46 mM potassium persulfate; the mixture was maintained at room temperature, in the dark, for 18 h before use. For the DPPH assay, the stable free radical DPPH (1,1-diphenyl-2-picrylhydrazyl) was dissolved in ethanol at a concentration of 0.16 mM, following the method described by Citta and co-authors [20]. Both assays are based on the reaction of the respective radical with antioxidant compounds, resulting in a decrease in absorbance. Peptides at concentrations of 0.05 and 0.1 mg/mL were mixed with either 0.08 mM ABTS^•+^ or 0.16 mM DPPH. The decrease in absorbance was measured spectrophotometrically at 415 nm and 517 nm for ABTS and DPPH, respectively, using a plate reader (Infinite M200 PRO, Tecan, Männedorf, Switzerland). For the ABTS assay, a calibration curve was set up with Trolox C, and the results are expressed as Trolox C equivalent antioxidant capacity (TEAC). For the DPPH assay, antioxidant activity was calculated as a percentage with respect to the control:

DPPH scavenging activity (%) = (Abs sample/Abs control) × 100

where Abs control is the absorbance of the DPPH solution alone, without any added compound, and Abs sample is the absorbance of DPPH in the presence of the peptide.

### 2.5. Cell Culture

Two colon cancer cell lines, Caco-2 and HCT116, were maintained as adherent cultures at 37 °C in a humidified atmosphere containing 5% CO_2_. Both cell lines were cultured in high-glucose Dulbecco’s Modified Eagle’s Medium (DMEM) supplemented with GlutaMAX, 10% fetal calf serum, and 1% Penicillin–Streptomycin (Thermo Fisher Scientific, Waltham, MA, USA).

### 2.6. Cell Viability

Cells were plated at a density of 1 × 10^4^ cells/well in 96-well plates and, after 48 h, exposed to the peptides at a final concentration of 0.05 mg/mL. Six hours post-treatment, oxidative stress was induced by the addition of 150 µM tert-butyl hydroperoxide (TbOOH). After 24 h from peptide addition, the culture medium was aspirated and replaced with 3-(4,5-dimethylthiazol-2-yl)-2,5-diphenyltetrazolium bromide (MTT) solution (0.5 mg/mL in phosphate-buffered saline, PBS), which was incubated for 3 h at 37 °C in the dark. The MTT solution was then removed, and formazan crystals were dissolved by adding 100 µL/well of isopropanol/DMSO (9:1). Absorbance was recorded at 595 and 690 nm using a microplate reader (Tecan Infinite^®^ M200 PRO, Männedorf, Switzerland) [12].

### 2.7. Estimation of ROS Production

Intracellular ROS production was assessed using the fluorescent probe 5-(and 6)-chloromethyl-2′,7′-dichlorohydrofluorescein diacetate (CM-H2DCFDA, Molecular Probes, Thermo Fisher Scientific, Waltham, MA, USA) to monitor H_2_O_2_ accumulation [12]. Cells were seeded at a density of 5 × 10^3^ cells/well in 96-well plates and allowed to grow for 48 h before treatment with peptides at 0.05 mg/mL for 24 h. Cells were then washed with Hank’s Balanced Salt Solution (HBSS) supplemented with 10 mM glucose and incubated with 10 µM CM-H2DCFDA for 20 min at 37 °C in the dark. Following a washing step with 100 µL/well of HBSS/10 mM glucose, oxidative stress was induced by exposure to 200 µM TbOOH. The increase in fluorescence was monitored for 90 min at an excitation wavelength of 485 nm and emission wavelength of 527 nm using a microplate reader (Tecan Infinite^®^ M200 PRO, Männedorf, Switzerland).

### 2.8. Evaluation of Lipid Peroxidation

Lipid peroxidation was evaluated as previously described [21]. Briefly, 4.5 × 10^5^ cells were plated in 6-well plates and exposed to peptides at 0.05 mg/mL after 48 h. Following 24 h of treatment, oxidative stress was induced by adding 300 µM TbOOH for 3 h. Cells were subsequently washed with 1 mL PBS (1×) and incubated with a mixture of 1 mL 0.1 N H_2_SO_4_ and 150 µL 10% phosphotungstic acid. Samples were centrifuged twice at 15,800× *g* for 10 min, and the resulting dry pellets were resuspended in 350 µL of a solution containing 0.25% NONIDET P-40, 0.01% butylated hydroxytoluene (BHT), and 0.25% thiobarbituric acid in H_2_O/acetic acid (1:1). After incubation at 95 °C for 60 min, cooled samples were centrifuged at 15,800× *g* for 10 min. The supernatants were mixed with 400 µL n-butanol, vigorously vortexed, and centrifuged again at the same speed for 15 min. The upper phase containing thiobarbituric acid reactive substances (TBARS) was quantified fluorimetrically at 530 nm (excitation) and 590 nm (emission) using a microplate reader (Tecan Infinite^®^ M200 PRO, Männedorf, Switzerland). The pellets were washed with 500 µL acetone/HCl 1 M (98:2) for 10 min at 4 °C, centrifuged at 15,800× *g* for 10 min at 4 °C, and dissolved in 75 µL RIPA lysis buffer containing 150 mM NaCl, 1% Triton X-100, 0.1% SDS, 0.5% DOC, 1 mM NaF, 1 mM EDTA, and 5 mM Tris/HCl (pH 7.4). Protein concentration was determined using the Lowry assay [22] for data normalization.

### 2.9. Total Glutathione and GSSG Estimation

Cells (4.5 × 10^5^) were seeded in a 6-well plate and, after 48 h, treated with peptides at a concentration of 0.05 mg/mL. After 6 h, 150 μM TbOOH was added to induce oxidative stress. Then, 24 h after peptide addition, cells were washed with 1 mL of PBS (1×) and promptly deproteinized with 2 mL of 6% *meta*-phosphoric acid. Following 20 min incubation on ice, cells were scraped, collected and centrifuged at 15,800× *g* for 10 min at 4 °C, and the resulting supernatants were neutralized with 15% Na_3_PO_4_ for total glutathione quantification. Sample aliquots were combined with 0.2 mM NADPH and 0.4 units of glutathione reductase (Sigma-Aldrich, St. Louis, MO, USA) in 0.2 M Na–K–Pi buffer (pH 7.4) containing 5 mM EDTA. The enzymatic reaction was initiated by the addition of 0.25 mM 5,5′-dithiobis(2-nitrobenzoic acid) (DTNB), and the resulting absorbance change was monitored at 412 nm for approximately 10 min using a Lambda 2 spectrophotometer (PerkinElmer, Waltham, MA, USA) [23]. For oxidized glutathione quantification, samples were treated with 2% 2-vinylpyridine for 40 min before performing the assay to derivatize reduced glutathione and quantify only oxidized glutathione [21]. The nanomoles of glutathione were calculated using a standard curve. The pellet obtained from the first centrifugation was washed with acetone for 30 min, centrifuged at 15,800× *g* for 10 min at 4 °C, dissolved in 150 μL of ice-cold RIPA lysis buffer, and analyzed using the Lowry assay [22] for protein normalization.

### 2.10. Western Blot Analysis of Nrf2 and Antioxidant Enzymes Expression in Treated Cells

Cells seeded in a 6-well plate (4 × 10^5^ cells/well) were treated with 0.05 mg/mL of the peptides. After 24 h, cells were collected, rinsed with 1 mL of PBS, and then lysed with 150 μL of ice-cold RIPA lysis buffer supplemented with 0.1 mM PMSF and protease inhibitor cocktail (Complete, Roche^®^, Basel, Switzerland) for 40 min on ice. After protein estimation, cell lysates (25 μg of proteins) were subjected to SDS-PAGE (4–12%), then blotted onto a 0.22 μm nitrocellulose membrane, which was subsequently blocked with 3% BSA in Tris-Buffered Saline (TBS) (50 mM Tris, 150 mM NaCl, pH 7.5). The membranes were probed with the selected primary antibodies: nuclear factor erythroid 2-related factor 2 (Nrf2, sc-365949, Santa Cruz biotechnology, Dallas, TX, USA), glutamate-cysteine ligase catalytic subunit (γ-GCSc, sc-390811), NAD(P)H Quinone Dehydrogenase 1 (NQO1, sc-32793), peroxiredoxin 1 (Prx1, sc-137222) and β-actin (PA0148, Abfrontier, Baileys Harbor, WI, USA). All primary antibodies were diluted 1:500 in 1% BSA in TBS and incubated overnight at 4 °C with orbital shaking. Western blot (WB) detection was carried out using UVITEC equipment (Alliance Q9 Advanced, Cambridge, UK), and densitometric quantification was performed using NineAlliance software (Mini 9 17.01 version, Uvitec Alliance, Cambridge, UK).

### 2.11. Assessment of Mitochondrial Respiration in Treated Cells

Mitochondrial respiratory function was assessed using the Seahorse XFe24 Analyzer (Agilent Technologies, Santa Clara, CA, USA) according to the Cell Mito Stress Test protocol. Caco-2 cells were plated at a density of 2 × 10^5^ cells/well in complete medium and treated with peptides at 0.05 mg/mL for 24 h. Prior to the oxygen consumption rate (OCR) measurements, the culture medium was exchanged with XF DMEM Assay Medium (pH 7.4) containing 10 mM glucose, 1 mM sodium pyruvate, and 2 mM glutamine, and the analysis was conducted at 37 °C. Three basal respiration measurements were recorded, followed by sequential injections of 1 µM oligomycin, 0.5 µM carbonyl cyanide-p-trifluoromethoxyphenylhydrazone (FCCP), and a combination of 1 µM antimycin A and 1 µM rotenone, with a 2 min mixing interval between each measurement [12]. Following the assay, cells were lysed in 50 µL RIPA buffer, and protein concentration was determined for data normalization.

### 2.12. Transepithelial Transport of Peptides Through Caco-2 Cell Monolayers

The intestinal barrier crossing capacity of the peptides was evaluated using differentiated Caco-2 cells [24]. Briefly, 4 × 10^4^ Caco-2 cells were seeded onto Transwell^®^ inserts (0.4 μm pore sizes, 12 mm diameter, 1.12 cm^2^ grown surface; Corning Life Sciences, Tewksbury, MA, USA), and their spontaneous differentiation in 21 days of culturing led to the formation of a monolayer. The transepithelial resistance (TEER) was analyzed via a Millicell ERS-2 volt-ohmmeter (EDM Millipore, Darmstadt, Germany) every 3 days to check for the formation of a complete monolayer that delimited an upper part (apical compartment) and lower part (basolateral compartment). The day of the experiment, the cell monolayer was rinsed three times with Hank’s balanced salt solution (HBSS) containing 10 mM D-glucose and equilibrated for 30 min at 37 °C. Afterward, the peptides (0.1 mg/mL final concentration) were added to the apical compartment in 0.7 mL HBSS at 37 °C. Samples collected from both compartments (apical and basolateral) at 10 and 120 min from peptide addition were centrifuged at 11,600× *g*, then frozen and lyophilized. Then, apical and basolateral fractions were resuspended and analyzed by Reversed Phase-High Performance Liquid Chromatography (RP-HPLC) using an Onyx monolithic C18 LC column 100 mm × 4.6 mm (Phenomenex, Torrance, CA, USA) with a linear gradient from 5% to 40% ACN with a flow rate of 2 mL/min. The peptide abundance was determined by monitoring the UV absorbance (λ = 220 nm) and then calculating the area under the curve of the different fractions collected at 10 and 120 min.

### 2.13. Statistical Analysis

Data are expressed as mean ± standard deviation (SD) of a minimum of three independent experiments. Statistical differences were evaluated via a one-way analysis of variance (ANOVA) followed by the Tukey–Kramer post hoc test for multiple comparisons, using GraphPad InStat 3 software. Differences were considered statistically significant at *p* < 0.05.

## 3. Results

### 3.1. Peptide Selection

Starting from the previously reported analysis of the composition of the peptide fraction obtained from milk permeate [12], we focused on the 5–30% acetonitrile peptide fraction, which was the one endowed with the best antioxidant activity and thus containing the most active antioxidant peptides. Of the 119 peptides identified in this fraction with a length between 7 and 14 amino acids, we focused on the ones derived from whey proteins. For further selection, an in silico approach was utilized. We analyzed which peptides could influence the Nrf2/Keap1 antioxidant pathway by examining the potential interaction of the peptides with the Kelch domain of Keap1, which is responsible for Nrf2 binding. A score was assigned to each peptide based on the probability of the interaction, taking into consideration different parameters such as the amino acid involved, the dissociation energy, and the overall quality of the predicted complex. The affinity of the different peptides for the target protein domain is shown in Table 1.

**Table 1:** List of the selected peptides. Acronym, sequence, length, protein of origin, solvation free energy gain upon formation of the docking assembly, and Peptide Ranker score are reported.

Acronym	Sequence	Lenght	Parent Protein	ΔG (kcal/mol)	Score (Peptide Ranker)
P10L	PLSILKEKHL	10	Glycosylation-dependent cell adhesion molecule 1	−5.9	0.278104
L11A	LPAPELGPRQA	11	Protein canopy homolog 3	−5.2	0.44036
L11I	LPIIQKLEPQI	11	Perilipin	−5.5	0.16865
N12L	NGQVWEESLKRL	12	Lactoperoxidase	1.1	0.602153
A13A	ALPIIQKLEPQIA	13	Perilipin	−7.4	0.269016
S13E	SQNPKLPLSILKE	13	Glycosylation-dependent cell adhesion molecule 1	−9.3	0.475187
L14K	LIVTQTmKGLDIQK	14	Beta-lactoglobulin	−6.6	0.074287
E14R	EGQEQEGEEmAEYR	14	Butyrophilin subfamily 1 member A1	−5.9	0.105524

**Figure 1:**
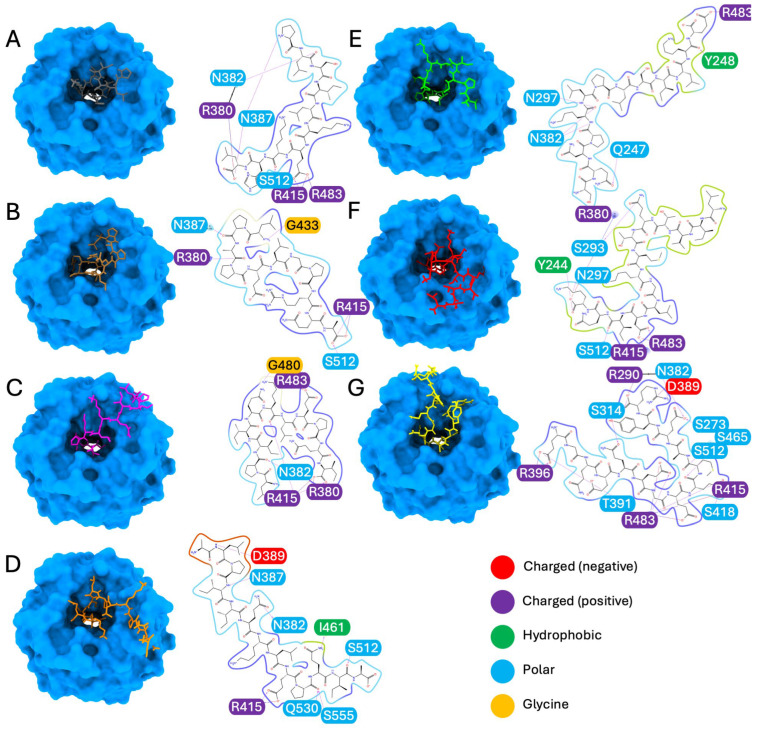
Molecular docking of the peptide–Keap1 complexes. The figure illustrates the docking poses of the following peptides with Keap1: (**A**) P10L, (**B**) L11A, (**C**) L11I, (**D**) A13A, (**E**) S13E, (**F**) L14K and (**G**) E14R. The binding interactions are represented at the level of the Kelch domain of Keap1 (light blue), showing both the binding geometry and the corresponding protein–peptide interaction diagrams. Amino acid residues are color-coded as follows: negatively charged (red), positively charged (blue), hydrophobic (light green), and polar (light blue).

For the seven top-ranking peptides (P10L, L11A, L11I, A13A, S13E, L14K, and E14R), the result of the molecular docking analysis is reported in Figure 1, providing valuable insights into their binding geometries and the nature of their protein–peptide interactions. More specifically, P10L forms hydrogen bonds with asparagine 382; asparagine 387; serine 512 and salt bridges with arginine 380, arginine 415 and arginine 483 (Figure 1A). L11A forms hydrogen bonds with arginine 380, asparagine 387, glycine 433 and serine 512, and a salt bridge with arginine 415 (Figure 1B). L11I forms hydrogen bonds with arginine 380, asparagine 382, glycine 480 and arginine 415, with which it also forms a salt bridge, as with arginine 380 (Figure 1C). A13A forms hydrogen bonds with asparagine 382, asparagine 387, aspartic acid 389, isoleucine 461, serine 512, glutamine 530, serine 555 and a salt bridge with arginine 415 (Figure 1D). S13E forms hydrogen bonds with glutamine 247, tyrosine 248, asparagine 297, asparagine 382, and a salt bridge with arginine 483 (Figure 1E). L14K forms hydrogen bonds with glutamine 244, serine 293, asparagine 297, serine 512, and salt bridges with arginine 380, arginine 415 and arginine 483 (Figure 1F). E14R forms hydrogen bonds with serine 273, arginine 290, serine 314, asparagine 382, aspartic acid 389, arginine 396, threonine 391, serine 415, serine 418, serine 465, and serine 512; salt bridges with arginine 415 and arginine 396; and multiple salt bridges and hydrogen bonds with arginine 483 (Figure 1G). Overall, these peptides appear to preserve the canonical Nrf2 anchoring mode, as they engage the key Keap1 arginine residues 380, 415 and 483 that also mediated recognition of the native Nrf3 ETGE motif. However, they also contact neighboring residues, reflecting a subtle adjustment in the binding pose within the Kelch pocket.

In the in silico analysis, we selected the following eight peptides for the subsequent analysis in cell models: P10L, L11A, L11I, N12L, A13A, S13E, L14K and E14R. As one can notice, seven of them were the ones with the best interaction probability (Figure 1 and Table S1) while N12L was chosen as it showed low affinity but had the best-ranking potential to be bioactive among our list using PeptideRanker software (https://peptide.ucd.ie/peptideranker/) [25] (accessed on October 2025), as reported in Table 1 and Table S2 of the Supplementary Materials. In addition, N12L was prioritized based not only on its high PeptideRanker score but also on its structural features that are relevant to antioxidant function. In particular, N12L contains a tryptophan (W), an aromatic amino acid that has been associated with enhanced radical-scavenging activity in peptides and proteins [26,27]. These properties may contribute to biological activity independent of Keap1 binding affinity and justify further experimental evaluation of N12L over other candidates.

### 3.2. Effect of the Peptides as Free Radical Scavengers

Our analysis of the synthetic peptides started with the determination of their potential antioxidant effect by evaluating their free radical scavenging ability. We employed two distinct methods for this purpose: the 2,2′-azinobis(3-ethylbenzothiazoline 6-sulfonate (ABTS) assay and the 1,1-diphenyl-2-picrylhydrazyl (DPPH) assay.

**Figure 2:**
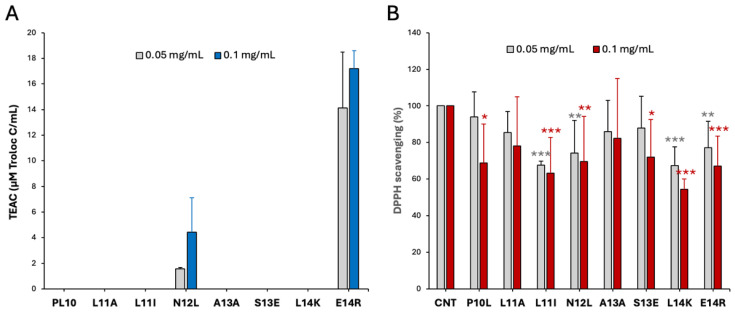
Antioxidant activity of the different peptides. The antioxidant activity was tested with 0.05 and 0.1 mg/mL of each peptide. (**A**) ABTS^•+^ scavenging activity is reported as Trolox *c* equivalent antioxidant capacity (TEAC); (**B**) DPPH scavenging assay, where results are reported as percentage with respect to the control (CNT); results are the mean +/− SD of 3 replicates, * *p* < 0.05; ** *p* < 0.01; *** *p* < 0.001.

The results, presented in Figure 2, show that the peptides N12L and E14R exhibit a strong capacity to scavenge both radicals in a concentration-dependent manner, with particularly high effectiveness against ABTS^•+^ (Figure 2A). Among the other peptides, L11I and L14K display antioxidant activity in the DPPH assay (Figure 2B), and P10L and S13E showed limited DPPH scavenging activity only at the higher concentration, while the remaining peptides were completely ineffective. Notably, these findings do not contradict the in silico analysis, as direct radical scavenging activity and Nrf2 targeting represent two distinct mechanisms for inducing an overall antioxidant effect.

### 3.3. Effect of the Peptides on Cellular Viability, ROS Production and Lipid Peroxidation

Next, we evaluated the antioxidant efficacy of the peptides in a cellular environment using two different cell lines, Caco-2 and HCT116, to obtain robust and reliable data. Both lines are derived from human colorectal carcinoma and are widely used as models for intestinal cells.

**Figure 3:**
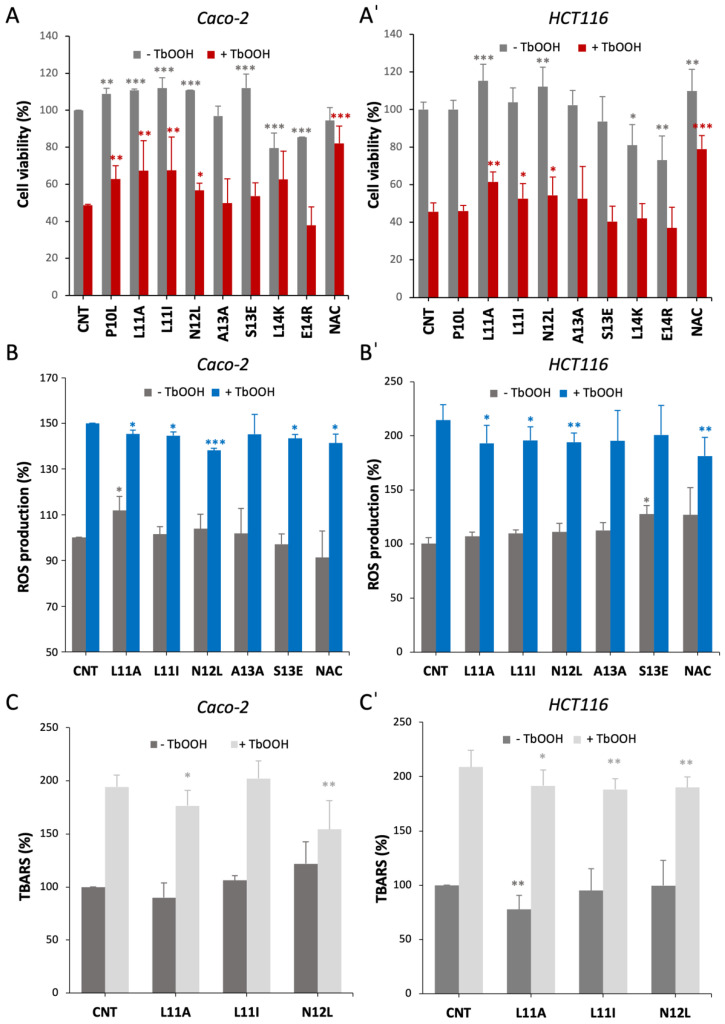
Protective effect of the selected peptides on oxidative stress induction in Caco-2 and HCT116 cells. (**A**,**A**’) Impact of peptides on cell viability and ROS production. Cells were exposed to the indicated peptides for 24 h, and oxidative stress was induced by the addition of 150 µM TbOOH for 18 h. (**A**) Caco-2 and (**A**’) HCT116 cell viability is expressed as percentage relative to the untreated control (CNT). (**B**) Caco-2 and (**B**’) HCT116: intracellular ROS levels were measured in peptide-treated cells in the absence (gray) or presence (blue) of 200 µM TbOOH and are reported as percentage relative to the untreated control (CNT). (**C**,**C**’) Effects of the peptides on cellular lipid peroxidation shown as percentage of TBARS in the absence (dark gray) or presence (light gray) of 150 μM TbOOH (**C**) Caco-2 cells and (**C**’) HCT116 cells; results are the mean ± SD of three replicates * *p* < 0.05; ** *p* < 0.01; *** *p* < 0.001.

We started by evaluating the effect of the peptides on cell viability. The treatment of Caco-2 cells (Figure 3A) and HCT116 cells (Figure 3A’) with the selected peptides for 24 h generally did not harm the cells apart from the two peptides of 14 amino acids, which decreased cell proliferation. In addition, some peptides even stimulated cell proliferation, as in the case of L11A and N12L, which are effective in both cell lines. Then, we analyzed potential cytoprotective effects of the peptides from a pro-oxidant molecule: *tert*-butyl hydroperoxide (TbOOH). The peptides L11A, L11I and N12L were able to partially protect the cells from the cytotoxic activity of TbOOH in both cell models (Figure 3(A,A’) red bars). This positive effect, albeit not as strong as the reference antioxidant molecule N-acetyl-cysteine (NAC), indicates the efficacy of these three peptides in contrasting oxidative stress. These data agree with what was observed in the DPPH assay (Figure 2B), in which these peptides exhibited an antioxidant effect.

In addition to assessing cell viability, we investigated the peptides’ antioxidant activity in cells by examining their influence on reactive oxygen species (ROS) production, both under basal conditions and after oxidative stress induced by TbOOH. Since the 14 aa-long peptides emerged as cytotoxic, we decided to exclude them from the subsequent experiments.

Under basal conditions, the treatment with the peptides did not significantly alter ROS levels compared to the control in both cell lines (Figure 3(B,B’), gray bars). As expected, TbOOH exposure increased ROS levels of approximately 1.5-fold in Caco-2 cells and 2-fold in HCT116 cells. Among the peptides tested, L11A, L11I, and N12L counteracted the oxidant’s effect, reducing ROS production in both cell models to a degree comparable to NAC (Figure 3(B,B’), blue bars). These findings align perfectly with the results observed in the cell viability assay.

As an additional measure of overall cellular oxidative damage, we assessed lipid peroxidation by determining the thiobarbituric acid reactive substances (TBARS). We focused on the three peptides that demonstrated antioxidant activity in previous experiments: L11A, L11I, and N12L. We analyzed their effect both under basal conditions and following TbOOH treatment (Figure 3(C,C’). In the absence of oxidative stimulus, the peptides generally did not affect lipid peroxidation, except for L11A, which slightly decreased levels in HCT116 cells. Interestingly, upon TbOOH addition, the peptides partially but significantly counteracted lipid peroxidation, except for L11I in Caco-2 cells. These results suggest that the inhibition of lipid peroxidation may not be the primary mechanism through which these peptides exert their antioxidant effect, but their action still partially preserves the lipid cell components from oxidation. We also determined the effect of the peptides on cellular glutathione levels in basal conditions and after TbOOH addition, as glutathione is one of the most abundant cellular antioxidant molecules involved in the protection from peroxidation; however, we did not find any difference with respect to the control (Figure S1). Consequently, we proceeded to investigate the impact of the peptides on the Keap1/Nrf2 axis.

### 3.4. Effect of the Peptides on Nrf2 Pathway

To elucidate the mechanism of action of the peptides, we investigated their potential effect on the Keap1/Nrf2 antioxidant signaling pathway. Under normal conditions, Nrf2 is typically confined to the cytoplasm in a complex with Keap1, which promotes its proteasomal degradation. However, oxidative stressors or electrophiles can trigger the dissociation of this complex, leading to the activation of Nrf2 activity, promoting the expression of antioxidant and Phase II enzymes [7]. In our previous research, we demonstrated that the peptide fraction isolated from milk permeate could induce the Nrf2 signaling cascade [12]. Building on this knowledge, we measured Nrf2 levels following cell treatment with our selected peptides.

**Figure 4:**
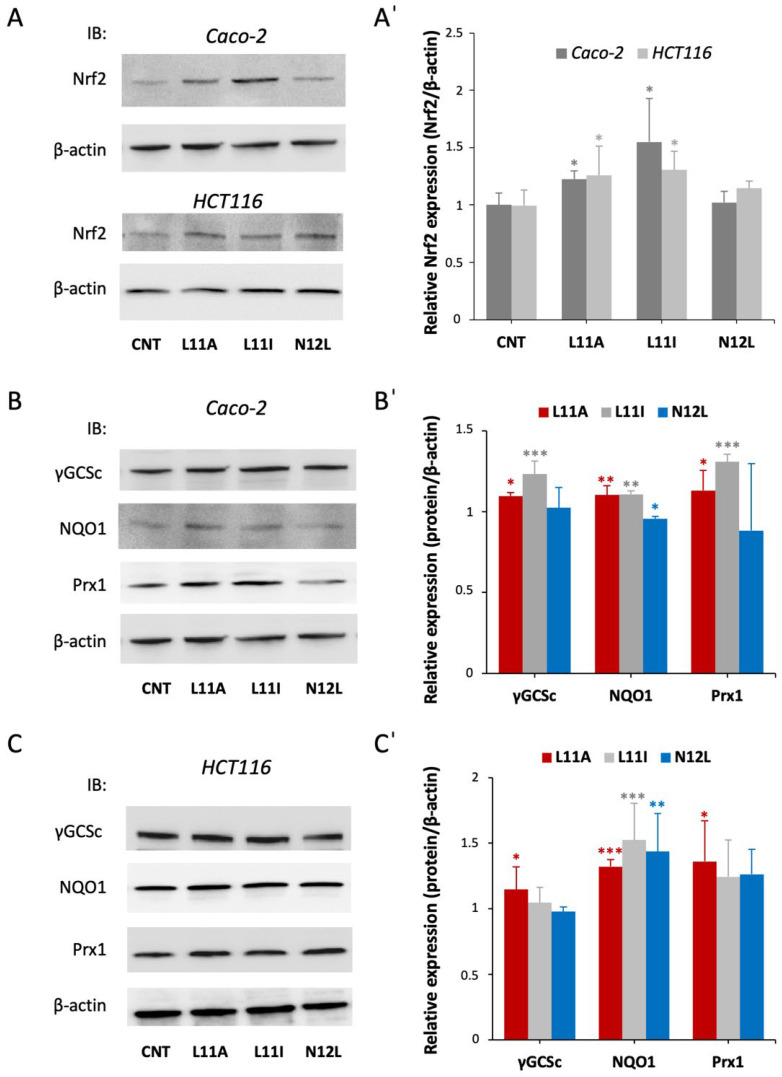
Effect of the peptides on Nrf2 activation and on the expression of downstream antioxidant enzymes. Cells were treated with the selected peptides for 24 h, proteins were extracted and Western blot analysis was carried out. (**A**) Representative WB of Nrf2 expression in Caco-2 and HCT116 cells; (**A**’) quantitative analysis of the WB after normalization using β-actin as a loading control. Results are the mean +/− SD of 3 replicates * *p* < 0.05. (**B**,**C**) Representative images of protein expression of the various antioxidant enzymes in Caco-2 (**B**) and HCT116 (**C**) cells via WB; (**B**’,**C**’) densitometric quantification of the Western blot bands normalized to β-actin as the housekeeping loading control. Results are the mean +/− SD of 3 replicates * *p* < 0.05; ** *p* < 0.01, *** *p* < 0.001. Of note, the β-actin blot shown in (**A**) for Caco-2 cells and (**B**) is the same, as both derive from the same experiment.

As reported in Figure 4(A,A’), the peptides L11A and L11I were able to increase the overall Nrf2 amount, suggesting a hindrance of Keap1-induced Nrf2 degradation in both cell lines. This evidence of Nrf2 activation is further supported by the increased expression of downstream antioxidant proteins (Figure 4B,C). In Caco-2 cells, L11I led to the strongest effect on Nrf2 activation, promoting a significant overexpression of γ-GCSc, NQO1 and Prx1, while the most effective peptide in HCT116 was L11A, which increased antioxidant gene expression.

Altogether, these results confirm the fact that the two peptides, L11A and L11I, can induce Nrf2 activation, as predicted in silico (Figure 1 and Table 1).

We also evaluated the effect of the peptides on cellular respiration utilizing the Caco-2 cell model to compare the effect of the isolated peptides with respect to what was observed previously with the peptide fraction [12]. As shown in Figure S2 of the Supplementary Materials, we found no differences in cells treated with the peptides with respect to the control apart from a slight increase in the basal respiration without effects on maximal or ATP-linked respiration in the presence of L11A. This result led us to the conclusion that mitochondrial function is not altered upon peptide addition.

### 3.5. Bioavailability of the Peptides Assessed via In Vitro Absorbance Simulation

An important feature that needs to be fulfilled for a molecule to exert bioactive effects in vivo is its capability to cross the intestinal barrier. For this reason, the intestinal absorption of the peptides was evaluated using Caco-2 cells grown in a Transwell^®^ diffusion system, where they differentiate into a monolayer mimicking the intestinal mucosa. This system separates each well into an apical compartment (resembling the intestinal lumen) and a basolateral compartment (facing the blood vessels), allowing simulation of intestinal absorption in a controlled setting [28]. Once the epithelium was formed, the purified peptides were loaded onto the apical compartment, and their amount in both compartments was quantified at 10 and 120 min after loading.

**Figure 5:**
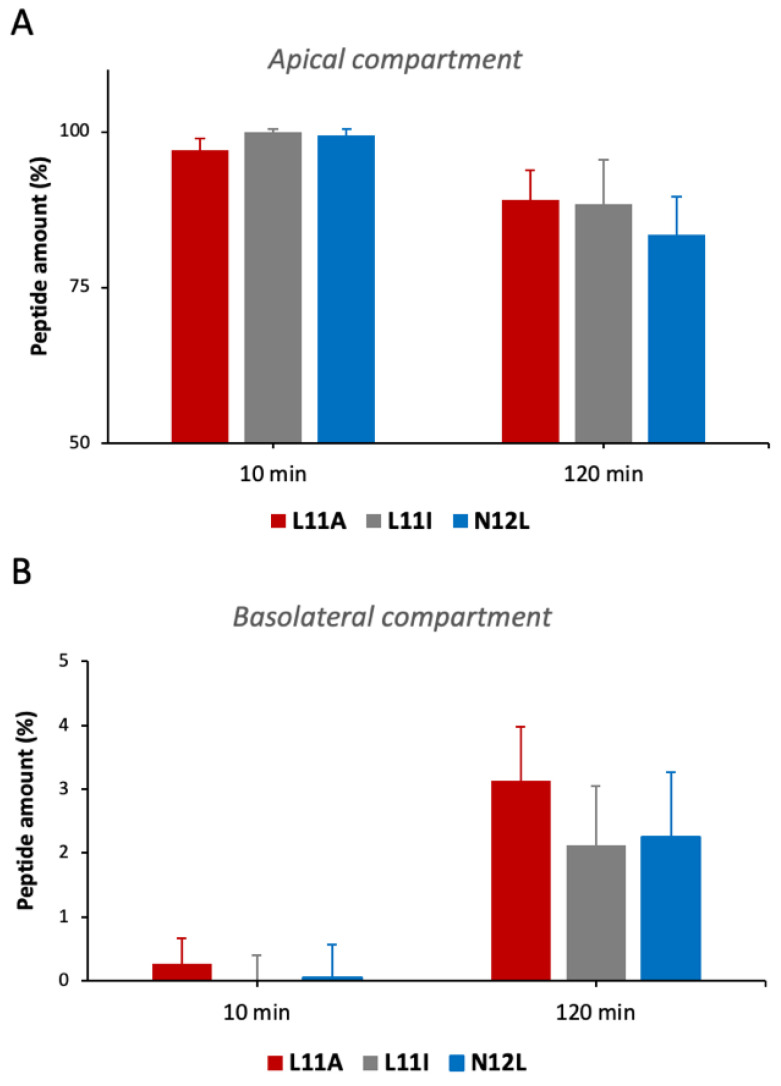
Quantification of the uptake of the peptides by the Caco-2 cell monolayer. Each of the three peptides was singularly administered to the apical compartment at time 0, then apical and basolateral compartments were collected after 10 and 120 min. RP-HPLC analysis was performed, and the amount of peptide in each fraction was quantified. (**A**) Amount of the peptides present in the apical compartment at 10 and 120 min; (**B**) amount of the peptides absorbed and detected in the basolateral side after 10 and 120 min.

Figure 5A shows that after 10 min, almost the entire amount of each peptide is still present in the apical compartment, while after 120 min, there is a significant decrease of about 10–18%. This reduction is comparable among the three peptides. Regarding the basolateral compartment (Figure 5B), L11A exhibits the best cell-crossing capacity, increasing from 0.3% after 10 min to 3.14% at 120 min. However, L11I and N12L also demonstrate the ability to pass the intestinal monolayer, both reaching similar levels of approximately 2% at 120 min. Thus, all three peptides are able not only to enter the intestinal epithelium but also to cross it and reach the basolateral compartment, suggesting a potential systemic effect.

## 4. Discussion

In this paper, a comprehensive analysis of the activity of peptides previously identified in milk permeate [12] revealed their multifaceted antioxidant properties, demonstrating efficacy both in vitro and in cellular environments. The analysis started from the peptide selection, which was performed using in silico screening. The structural analysis of the seven top-ranking peptides (P10L, L11A, L11I, A13A, S13E, L14K, and E14R) in complex with the Keap1-Kelch domain reveals a consistent pattern of interactions, particularly involving key arginine residues, Arg380, Arg415, and Arg483, located in the first two binding pocket subdomains. These residues form critical electrostatic contacts with the peptide’s polar motifs, mimicking the high-affinity ETGE motif of Nrf2 and thereby stabilizing the complex. This finding aligns closely with the in silico screening strategy employed previously to evaluate peptides’ potential to disrupt the Keap1/Nrf2 interaction and activate the Nrf2 antioxidant response pathway [19].

In addition, for N12L, the prediction of its potential bioactive activity has been performed based on an N-to-1 neural network: PeptideRanker [25]. In this regard, it is important to note that this tool does not provide a prediction of the degree of bioactivity but only the probability of a biological effect.

The selected peptides range from 10 to 14 amino acids in length. Most are predominantly composed of non-polar amino acids (L, I, A), except for S13E, which is rich in non-charged polar residues (S, P). Nearly all peptides contain both basic and acidic amino acids (Q, E), except P10L. Notably, N12L contains a tryptophan (W) residue, which was one of the reasons for its selection as already stated above.

Regarding the isoelectric point (pI), most peptides have a pI around 7 and are uncharged at neutral pH. Exceptions are P10L and S13E, which have a more basic pI and are thus positively charged, and E14R, which has an acidic pI and a net charge of −5, due to its high content of negatively charged residues (E).

On the first analyses in vitro, the peptides, particularly N12L and E14R, exhibited significant free radical scavenging abilities in ABTS^•+^ and DPPH assays, indicating their potential as direct antioxidants (Figure 2). It is important to understand that we cannot directly compare the results from the two scavenging tests because each uses a different type of radical subjected to specific rate equilibria in reacting with the peptides.

In cellular models using Caco-2 and HCT116 cell lines, some of the peptides demonstrated protective effects against oxidative stress. In particular, L11A, L11I and N12L effectively preserved cell viability (Figure 3A), reduced ROS production induced by TbOOH (Figure 3B) and partially mitigated lipid peroxidation, as evidenced by decreased MDA levels (Figure 3C). The use of two distinct cell lines, both derived from human colorectal carcinoma but with different characteristics, provided robust and reliable data, strengthening the validity of our findings. TbOOH is a well-known inducer of oxidative stress that generates free radicals, triggering an overproduction of ROS, which in turn attack polyunsaturated fatty acids in cell membranes, initiating a lipid peroxidation cascade reflected by a significant increase in MDA levels. Although the peptides only partially reduced lipid peroxidation, they effectively reduced ROS levels and preserved cell viability. Notably, N12L, which contains a tryptophan residue known for its radical scavenging properties, may exert its protective effect through direct free radical scavenging. In fact, tryptophan’s antioxidant properties are well-known due to the indole ring that effectively neutralizes various free radicals via electron or hydrogen atom transfer and can be influenced by peptide sequence, length, and tryptophan position [26,27].

The mechanisms underlying the protective effects of the other peptides appear to be more complex and indirectly mediated by intracellular pathways.

Previous research by our group revealed that milk-derived peptides and peptide fractions not only display direct antioxidant activities but also modulate oxidative stress pathways at the cellular level, suggesting their role in enhancing cellular defense mechanisms against oxidative damage [12,19]. Our study revealed that L11A and L11I act as antioxidants through the Keap1/Nrf2 pathway, a master regulator of the cellular antioxidant response (Figure 4A). We also observed increased expression of Nrf2-controlled antioxidant proteins (Figure 4B,C). These findings align with our in silico predictions, validating our screening method.

The fact that the longer peptides induced a lowering of cellular proliferation could be attributable to the fact that some peptides can insert into cell membranes, forming pores or disrupting the lipid bilayer, leading to cell death or to a tendency of these peptides to form aggregates [29,30,31].

Several in vitro studies have reported that short casein-derived peptides, including YQLD, FSDIPNPIGSEN, YFYP, and KVLPVPEK, exhibit measurable free radical scavenging activity and the ability to protect cells against oxidative insults induced by agents such as 2,2-azobis(2-methylpropylimid) dihydrochloride or TbOOH [19,32]. However, direct comparison of the antioxidant potency across studies remains challenging, given the considerable variability in peptide concentrations, oxidative stimuli, and cell lines employed in different publications.

Our permeability studies demonstrated that these peptides, above all L11A, can cross the intestinal monolayer (Figure 5). Regarding the way by which these peptides are absorbed, it is known that 10–15 amino acid-long peptides can enter enterocytes via pepT1-like transporters and the intracellular transcytosis pathway and/or can be paracellularly absorbed through the tight junctions [13]. These pathways collectively enable transport into the systemic circulation, with paracellular diffusion primarily favoring smaller, hydrophilic peptides through tight junctions and transcellular mechanisms contributing particularly for larger or more lipophilic sequences; recent insights emphasize how tight junction regulation and epithelial permeability determine paracellular flux, while cellular pathways govern transcellular movement [13,33,34].

The intestine cell-crossing capacity of the peptides is particularly noteworthy, as it suggests that these peptides could potentially exert their antioxidant effects beyond the gut, reaching other tissues via systemic circulation. The ability of bioactive peptides to cross the intestinal barrier is a crucial factor in their potential therapeutic applications, as it allows for systemic effects rather than just local action. This aligns with emerging research on bioactive peptides and their potential for wide-ranging health benefits [3,9].

The differences in the peptides’ mechanism of action highlight the complexity of their antioxidant action and suggest that a combination of these peptides might provide a more comprehensive antioxidant effect. The partial but significant counteraction of lipid peroxidation by these peptides, particularly evident after TbOOH treatment, adds another layer to their antioxidant profile. However, the fact that this effect was not as pronounced as their impact on ROS levels suggests that inhibition of lipid peroxidation may not be the primary mechanism of their antioxidant action. This underscores the importance of our subsequent investigation into the Keap1/Nrf2 axis and highlights the multifaceted nature of cellular antioxidant defense mechanisms.

While in vitro in nature, these results are a promising stepping stone toward future validation in animal models and clinical settings. Furthermore, despite the acknowledged limitation that the described antioxidant mechanisms may not fully capture the complexity of the observed activity, the ability to recover bioactive peptides from a by-product fraction underscores the significance of our results.

## 5. Conclusions

In conclusion, this study demonstrates that the peptides L11A, L11I, and N12L derived from milk permeate possess significant multifunctional antioxidant properties, inducing protection against cellular oxidative stress through direct free radical scavenging for N12L and by activating the Keap1/Nrf2 pathway for L11A and L11I. Their ability to cross the intestinal barrier further supports their potential as nutraceuticals or therapeutic agents targeting oxidative stress-related conditions. Notably, as these peptides are naturally present in milk permeate, they represent a significant valorization of this by-product. Future studies should focus on in vivo models to further validate these promising results, assess bioavailability and explore the potential incorporation of these bioactive peptides into functional foods as a strategy to prevent or reduce oxidative stress-related conditions.

## Data Availability

Data is contained within the article or Supplementary Materials.

## Supplementary Materials

The following supporting information can be downloaded at: https://www.mdpi.com/article/10.3390/antiox15050527/s1, Table S1: Peptides were analyzed in silico for their affinity with the Kelch domain of Keap1. Solvation free energy and dissociation energy, alongside the solvation energy *p*-value, are reported. Table S2: Scores of the various peptides obtained with the online tool Peptide Ranker. Figure S1: Quantification of total glutathione and glutathione dimer amount in cells treated with the peptides. Figure S2: Oxygen consumption rates of Caco-2 cells treated with the peptides.

## Abbreviations

The following abbreviations are used in this manuscript:

ABTS	2,2′-azinobis(3-ethylbenzothiazoline 6-sulfonate).
CM-H_2_DCFDA	5-(and 6)-chloromethyl-20,70-dichlorohydrofluorescein diacetate.
DPPH	1,1-diphenyl-2-picrylhydrazyl.
γ-GCSc	Glutamate-cysteine ligase catalytic subunit.
Keap1	Kelch-like ECH-associated protein 1.
MTT	3-(4,5-dimethylthiazol-2-yl)-2,5-diphenyltetrazolium bromide.
NQO1	NAD(P)H Quinone Dehydrogenase 1.
Nrf2	Nuclear factor erythroid 2-related factor 2.
Prx1	Peroxiredoxin 1.
RP-HPLC	Reversed-phase high-performance liquid chromatography.
ROS	Reactive oxygen species.
TBARS	Thiobarbituric acid reactive substances.
TbOOH	*tert*-butyl hydroperoxide.
TEAC	Trolox C equivalent antioxidant capacity.
