# Supplementary material for: From By-Product to Bioactive: New Antioxidant and Bioavailable Peptides Derived from Milk Permeate Targeting the Nrf2/Keap1 Pathway in Intestinal Cell Models
Source: Antioxidants (Basel). 2026 Apr 22;15(5):527. doi: 10.3390/antiox15050527 (PMC13203221; doi:10.3390/antiox15050527)
Supplement: Supplementary file 1 [file antioxidants-15-00527-s001.zip › SM Scalcon V. et al..pdf]

Supplementary material

From Byproduct to Bioactive: New Antioxidant and Bioavailable Peptides Derived from Milk Permeate Targeting Nrf2/Keap1 Pathway in Intestinal Cell Models

Valeria Scalcon,[1*] Alessandro Grinzato,[1] Federico Fiorese,[1] Alessandra Folda,[1] Stefania Ferro,[1] Gianfranco Betti,[2] Marco Bellamio,[3] Emiliano Feller,[3] Oriano Marin,[1] Maria Pia Rigobello.[1#]

[1] Department of Biomedical Sciences, University of Padova, via Ugo Bassi 58/b, 35132, Padova, Italy

[2] Centrale del latte d'Italia S.p.A., sede di Firenze, via dell'Olmatello 20, 50127, Firenze, Italy

[3] Centrale del latte d'Italia S.p.A., sede di Vicenza, via Faedo 60, 36100, Vicenza, Italy

[*] **corresponding Authors:** Valeria Scalcon valeria.scalcon@unipd.it

Maria Pia Rigobello mariapia.rigobello@unipd.it

Table S1. Peptides were analyzed *in silico* for their affinity with the Kelch domain of Keap1. Solvation free energy and dissociation energy, alongside with the solvation energy P-value are reported.

Acronym	Sequence	Lenght	Parent protein	ΔG int (kcal/mol)	ΔG dis (kcal/mol)	P-value
G9K	GQVWEESLK	9	lactoperoxidase	-2.6	-1.2	0.653
D10R	DRITGGKDFR	10	Liproprotein lipase	-4.2	-2.1	0.216
P10L	PLSILKEKHL	10	Glycosylation-dependent cell adhesion molecule 1 (GLCM1)	-5.9	3.2	0.709
I10R	ILWESASLLR	10	Complement C3	-3.1	-0.2	0.777
Y10R	YDDRNEPVLR	10	Mucin 15	-2.3	0.1	0.345
A11R	AAPAGAAIQSR	11	Polimeric immunoglobulin receptor	-1.7	-2.4	0.649
Y11T	YSGSSKALVST	11	Polimeric immunoglobulin receptor	-3.4	-1.4	0.699
L11A	LPAPELGPRQA	11	Protein canopy homolog 3	-5.2	2.6	0.369
Q11K	QPTNQVVANAK	11	perilipin	-4.8	1.8	0.19
Q11L	QPQSQNPKLPL	11	GLCM1	-4.4	-0.9	0.528
L11L	LPLSILKEKHL	11	GLCM1	-5	-1.9	0.62
L11I	LPIIQKLEPQI	11	perilipin	-5.5	2.7	0.474
Y12L	YSGSSKALVSTL	12	Polimeric immunoglobulin receptor	-4.1	-1.3	0.753
A12S	ALLDPSFFAKES	12	Polimeric immunoglobulin receptor	-3.1	-0.1	0.695
L12A	LPIIQKLEPQIA	12	perilipin	-2.7	-1	0.669
I12E	ILNKPEDETHLE	12	GLCM1	-3.4	-1.1	0.768
N12L	NGQVWEESLKRL	12	Lactoperoxidase	1.1	-1.9	0.701

A13S	AGEIQNKALLDPS	13	Polimeric immunoglobu lin receptor	-2.5	-1.2	0.642
V13R	VEDHIAEGSVAVR	13	Butyrophilin subfamily 1 member A1	-3.6	-1.8	0.603
D13R	DPARVLDLGPITR	13	Glycoprotein 2	-8.5	4.6	0.3
A13A	ALPIIQKLEPQIA	13	perilipin	-7.4	3.2	0.386
S13P	SSRQPQSQNPKLP	13	GLCM1	0.4	-4.1	0.733
S13E	SQNPKLPLSILKE	13	GLCM1	-9.3	3.3	0.244
A14L	AQPTDASAQFIRNL	14	GLCM1	-1.6	-1.6	0.565
S14L	SSRQPQSQNPKLPL	14	GLCM1	-2.9	-3.,7	0.35
L14K	LIVTQTmKGLDIQK	14	Beta-lactoglobulin	-6.6	1.7	0.421
T14K	TAIRNGQVWEESLK	14	Lactoperoxid ase	-2.4	-1.7	0.826
E14R	EGQEQEGEEmAEYR	14	Butyrophilin subfamily 1 member A1	-5.9	3.4	0.388

Table S2. Scores of the various peptides obtained with the online tool Peptide Ranker. Any peptide predicted over a 0.5 threshold is labelled as bioactive and the peptides are reported from the one ranked as the best to the worst.

Abbreviation	Peptide sequence	Score
N12L	NGQVWEESLKRL	0.602153
Q11L	QPQSQNPKLPL	0.600281
A14L	AQPTDASAQFIRNL	0.59253
S14L	SSRQPQSQNPKLPL	0.484745
S13E	SQNPKLPLSILKE	0.475187
I10R	ILWESASLLR	0.460712
A12S	ALLDPSFFAKES	0.445196
L11A	LPAPELGPRQA	0.44036
D13R	DPARVLDLGPITR	0.435884
L11L	LPLSILKEKHL	0.358655
S13P	SSRQPQSQNPKLP	0.350221
A11R	AAPAGAAIQSR	0.319971
P10L	PLSILKEKHL	0.278104
Y12L	YSGSSKALVSTL	0.275648
Y10R	YDDRNEPVLR	0.275521
A13A	ALPIIQKLEPQIA	0.269016
G9K	GQVWEESLK	0.235866
A13S	AGEIQNKALLDPS	0.234276
D13R	DRITGGKDFR	0.223131
L12A	LPIIQKLEPQIA	0.18014
L11I	LPIIQKLEPQI	0.16865
V13R	VEDHIAEGSVAVR	0.147976
Y11T	YSGSSKALVST	0.141465
E14R	EGQEQEGEEMAEYR	0.105524
T14K	TAIRNGQVWEESLK	0.103195
Q11K	QPTNQVVANAK	0.0933338
I12E	ILNKPEDETHLE	0.0931189
L14K	LIVTQTMKGLDIQK	0.074287

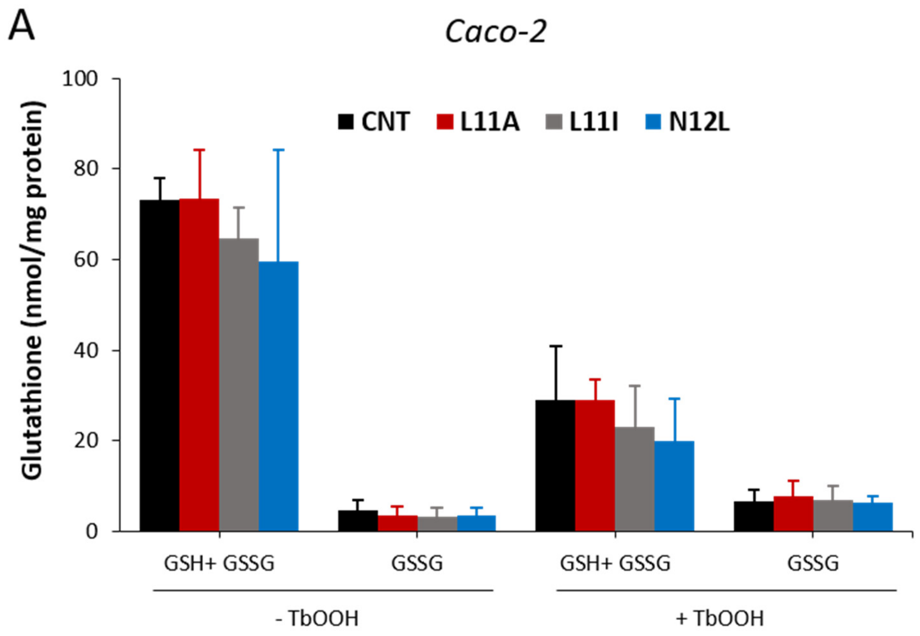

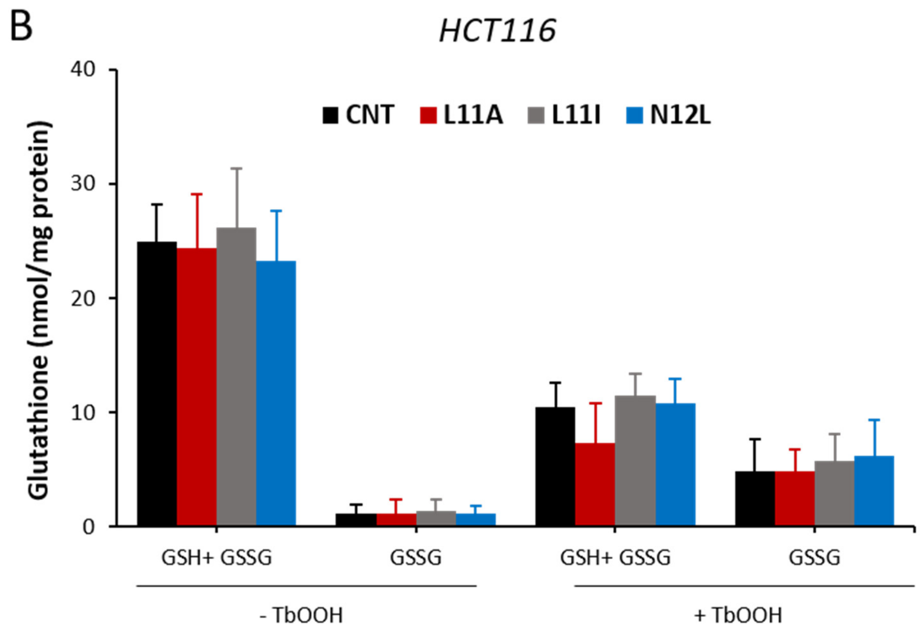

Figure S1. Quantification of total glutathione and glutathione dimer amount in cells treated with the peptides. Cells were treated with the peptides (0.05 mg/mL) and 150 μM TbOOH was added after 6 h. After 24 h from peptide treatment, cells were processed and glutathione amount quantified as reported in the Materials and Methods section. Data are reported as nmoles of GSH / mg proteins. **A)** Results obtained in Caco-2 cells; B) Results obtained in HCT116 cells. Results are the mean ± SD of 3 experiments, differences between groups are not significant ($p>0.05$).

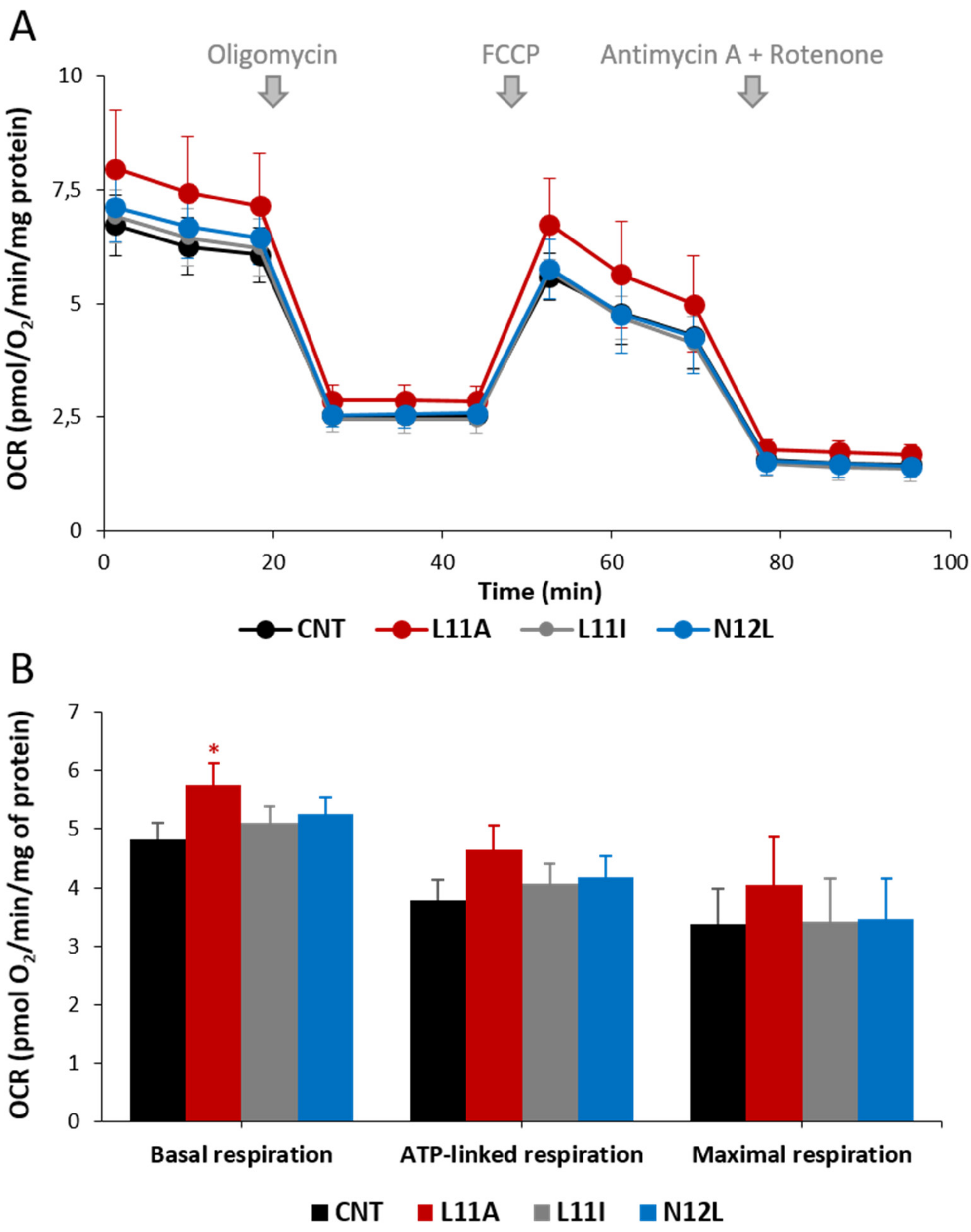

Figure S2. Oxygen consumption rates of Caco-2 cells treated with the peptides. Caco-2 cells were treated with 0.05 mg/mL of the peptides for 24 h. The oxygen consumption rates (OCRs) were assessed using the Seahorse Xfe24 analyzer as described in the Materials and Methods section. Basal respiration and respiratory capacity in the presence of sequential addition of 1 µM oligomycin, 0.5 µM FCCP and the combination of 1 µM antimycin A + 1 µM rotenone, was measured. **A)** OCR values normalized for the protein content; **B)** Basal, ATP-linked and maximal respirations are shown as the mean ± SD of 3 experiments, *$p < 0.05$, if not stated differences are not significant ($p>0.05$).
